# Psychospiritual Profiles Differentiate Dietary and Lifestyle Behaviors

**DOI:** 10.3390/nu18122007

**Published:** 2026-06-20

**Authors:** Sebastian Binyamin Skalski-Bednarz, Loren L. Toussaint, Magdalena Piegza, Monika Bidzan-Wiącek, Mariola Bidzan

**Affiliations:** 1 Institute of Psychology, Ignatianum University in Cracow, 31-501 Cracow, Poland; 2 Faculty of Philosophy and Education, Catholic University of Eichstätt-Ingolstadt, 85072 Eichstätt, Germany; 3 Department of Psychology, Luther College, Decorah, IA 52101, USA; touslo01@luther.edu; 4 Department of Psychiatry, Faculty of Medical Sciences in Zabrze, Medical University of Silesia in Katowice, 42-612 Tarnowskie Góry, Poland; mpiegza@sum.edu.pl; 5 Faculty of Health Sciences with the Institute of Maritime and Tropical Medicine, Medical University of Gdańsk, 80-210 Gdansk, Poland; monika.bidzan-wiacek@gumed.edu.pl; 6 Institute of Psychology, Faculty of Social Sciences, University of Gdansk, 80-309 Gdansk, Poland; mariola.bidzan@ug.edu.pl; 7 Institute of Pedagogy and Languages, University of Applied Sciences in Elbląg, 82-300 Elbląg, Poland

**Keywords:** psychospiritual profiles, dietary behaviors, latent profile analysis, religiousness, spirituality, psychological well-being

## Abstract

**Background/Objectives:** Previous literature has linked nutrition with both psychological distress and well-being. However, less is known about how psychological and spiritual resources cluster within individuals or whether distinct psychospiritual profiles are associated with dietary and lifestyle behaviors. This study examined these associations using a person-centered approach. **Methods:** A community sample of 522 adults from the United States completed measures of perceived stress, depressive symptoms, coping self-efficacy, gratitude, forgiveness, religiousness/spirituality, daily spiritual experiences, religious/spiritual meaning and beliefs, and dietary and lifestyle behaviors. Latent profile analysis was conducted to identify psychospiritual profiles. **Results:** Four profiles were identified: Moderate (*n* = 195), Flourishing (*n* = 199), Vulnerable (*n* = 70), and Maladaptive (*n* = 58). The Flourishing profile demonstrated the most adaptive psychological functioning and was associated with healthier dietary behaviors, including lower breakfast skipping and fast-food consumption, greater whole-grain and vegetable intake, lower salt use, and lower sweets and dessert intake. The Vulnerable profile demonstrated the highest levels of perceived stress and depressive symptoms together with relatively elevated religiousness/spirituality, whereas the Maladaptive profile was characterized by elevated distress and consistently low levels of psychological and spiritual resources. Overall, the Vulnerable and Maladaptive profiles demonstrated less favorable dietary patterns relative to the Flourishing and Moderate profiles. However, the observed effects were generally modest and selective. **Conclusions:** Dietary and lifestyle behaviors may be associated with broader psychospiritual configurations rather than isolated psychological characteristics alone. The findings additionally highlight the heterogeneous nature of religiousness and spirituality within psychological functioning.

## 1. Introduction

Research suggests that dietary and lifestyle behaviors are associated with psychological functioning and mental health. Healthier dietary patterns, including greater consumption of fruits, vegetables, and whole grains, are linked to lower levels of stress, depression, and anxiety, whereas unhealthy eating behaviors are associated with poorer psychological functioning [1,2]. Deficiencies in nutrients such as omega-3 fatty acids, magnesium, vitamin D, and B vitamins have additionally been associated with impaired emotional regulation and depressive symptoms [3,4,5]. Some studies also suggest that nutritional factors and gut microbiota may be related to mood, stress sensitivity, and cognitive functioning through gut–brain pathways [6,7,8]. Nevertheless, the relationships between nutrition and psychological functioning appear to be bidirectional, as psychological distress may also contribute to maladaptive eating behaviors and increased consumption of highly processed foods [9].

Large-scale meta-analytic evidence based on more than 119,000 adults indicates that higher stress levels are associated with increased consumption of unhealthy foods and reduced intake of healthy foods [10]. Specifically, stress has been linked to greater intake of highly palatable foods rich in fat and sugar and lower consumption of fruits and vegetables [11,12,13,14,15]. These associations may be explained by the reward-based stress eating model, which proposes that stress-related activation of neuroendocrine and reward systems increases the salience of highly palatable foods [16].

Beyond associations with stress and psychopathology, dietary behaviors have also been linked to positive dimensions of mental health, as higher consumption of fruits and vegetables has been associated with greater happiness, life satisfaction, positive affect, and eudaimonic well-being, including greater feelings of meaning, engagement, curiosity, and creativity [17,18,19,20]. Similarly, evidence from eating disorder research suggests that well-being and psychopathology may represent related but distinct dimensions of functioning, as some individuals with elevated eating disorder symptoms may still report relatively high levels of flourishing and psychosocial functioning [21,22].

Importantly, associations between psychological functioning and eating behaviors appear to vary substantially across individuals and may be associated with differences in emotional regulation, eating styles, coping-related processes, and broader psychological functioning [9,23,24,25]. However, despite growing evidence linking nutrition with both psychological distress and flourishing, the role of broader positive psychological resources in dietary and lifestyle behaviors remains relatively underexplored.

One psychological factor that has received attention in the context of health behaviors is self-efficacy. Within social cognitive theory [26], self-efficacy refers to beliefs about one’s capability to organize and execute behaviors necessary to achieve desired outcomes. Cross-sectional [27] and intervention-based [28] studies suggest that higher nutrition-related self-efficacy is associated with lower fat intake and higher consumption of fiber, fruits, and vegetables, while increases in self-efficacy over time may contribute to subsequent improvements in dietary behaviors.

Some interest has also been directed toward positive emotional resources such as gratitude. According to the broaden-and-build theory [29], positive emotions may expand cognitive and behavioral repertoires and facilitate the development of enduring psychological resources relevant to adaptive functioning. This perspective may also extend to health-related and dietary behaviors. In this context, gratitude has been associated with healthier eating behaviors and greater consumption of healthy foods among adolescents and young adults, while gratitude-based interventions have been linked to healthier eating partly through reductions in negative affect [30]. These findings suggest that positive emotional resources may influence eating-related behaviors partly through their associations with emotional functioning and stress regulation.

Forgiveness has also been conceptualized as an emotion-focused coping resource associated with lower maladaptive responses to stress [31]. Gratitude and forgiveness are additionally regarded as related positive psychological resources, as both have been associated with adaptive coping, interpersonal functioning, and broader well-being [32]. Recent evidence further suggests that higher forgiveness is associated with lower emotional eating and less adult weight gain, potentially through reduced reliance on maladaptive coping responses to emotional distress [33], indicating that forgiveness may also relate to healthier dietary and lifestyle behaviors.

Religiousness and spirituality may also be relevant to dietary and lifestyle behaviors, as many religious traditions include norms and practices related to eating, fasting, moderation, and self-discipline [34]. Contemporary positive psychology perspectives increasingly conceptualize religiousness and spirituality not only as protective factors against psychopathology, but also as potential determinants of flourishing, meaning in life, adaptive coping, and psychosocial well-being [35,36]. However, contemporary approaches also emphasize that religiousness and spirituality are not homogeneous constructs and may be associated with both beneficial and maladaptive outcomes depending on the specific forms of religious involvement and the ways religion or spirituality are experienced and expressed [37].

Systematic [38] and narrative [34] reviews indicate that greater religiousness and spirituality are generally associated with higher fruit and vegetable intake, whereas associations with fat intake, dietary quality, overweight, and obesity appear more mixed. Certain religious fasting practices, including Ramadan fasting, have additionally been associated with lower cholesterol levels, lower body weight, and improved metabolic indicators among healthy individuals and some cardiovascular patients [39]. At the same time, some highly restrictive dietary practices may increase the risk of nutritional deficiencies when not adequately balanced [40]. Similar heterogeneity has also been observed in the context of body image and eating disorders. More internalized religious beliefs and secure attachment to God have been linked to lower levels of disordered eating and body image concerns, whereas findings for religiousness and spirituality overall remain less consistent across studies [41,42].

Taken together, these findings may be understood within a broader relational perspective on spirituality. The relational spirituality model [43] conceptualizes religiousness and spirituality as embedded within individuals’ relationships with the self, others, communities, and the sacred, shaping coping, emotional regulation, meaning-making, and health-related functioning, while also contributing to both adaptive and maladaptive outcomes depending on the ways spirituality is experienced and expressed within interpersonal and cultural contexts [44]. In combination with the broaden-and-build theory [29], it may be suggested that dimensions of religiousness and spirituality, aligned with gratitude, forgiveness, and coping self-efficacy, may interact with one another as broader psychosocial resources associated with health, including dietary and lifestyle behaviors.

Although prior studies have examined isolated associations between nutrition and individual psychosocial variables, relatively little is known about how broader constellations of psychological and spiritual resources co-occur within individuals and whether such naturally occurring psychospiritual profiles are associated with dietary and lifestyle behaviors. Moreover, previous research has relied predominantly on variable-centered approaches, which may obscure heterogeneity in the ways positive psychological resources, distress, and dimensions of religiousness and spirituality coexist within individuals. These co-occurring dimensions may better reflect the lived complexity of psychosocial functioning, as individuals may simultaneously experience psychological distress, coping capacities, positive emotional resources, and spiritual orientations in different configurations. A person-centered approach is therefore useful for identifying naturally occurring patterns of risk and resource factors that may be differentially associated with dietary and lifestyle behaviors.

### Current Study

Given the multidimensional and potentially heterogeneous nature of psychological and spiritual resources, person-centered approaches such as latent profile analysis (LPA) may provide a useful framework for identifying naturally occurring configurations of psychosocial and spiritual functioning associated with dietary and lifestyle behaviors. Accordingly, the present study aimed to examine whether distinct profiles of psychospiritual functioning are associated with dietary and lifestyle behaviors. Because previous findings suggest that religiousness and spirituality are multidimensional constructs whose associations with health behaviors may vary depending on their specific forms and expressions [34,40], the study distinguished between several related but conceptually distinct dimensions of religious and spiritual functioning: self-perceived religiousness and spirituality, daily spiritual experiences, and religious/spiritual meaning, values, and beliefs.

To examine broader protective tendencies in a general population sample, the study focused primarily on relatively stable dispositional resources rather than situation-specific coping responses. Distress-related indicators (i.e., perceived stress and depressive symptoms) were additionally included in the profile modeling because previous findings suggest that psychological difficulties and positive functioning may coexist within individuals [21,22], allowing for the identification of more differentiated profiles reflecting both psychosocial strengths and vulnerability. Because the number and structure of potential latent profiles have not yet been clearly established in the literature, no a priori assumptions were made regarding the specific profile solution. Nevertheless, it was expected that profiles characterized by lower perceived stress and depressive symptoms, together with higher levels of coping self-efficacy, gratitude, forgiveness, and adaptive dimensions of religiousness and spirituality, would be associated with relatively healthier dietary and lifestyle behaviors, whereas profiles characterized by greater distress and lower psychosocial and spiritual resources would demonstrate relatively less adaptive dietary and lifestyle patterns.

## 2. Materials and Methods

### 2.1. Participants

The study was conducted as part of the Kellogg-funded Northeast Iowa Food and Fitness Initiative in collaboration with Luther College. Participants were recruited through community-based workplace and educational settings involved in the initiative, including local employment and educational contexts in Northeast Iowa. Potential respondents were approached through these participating settings and invited to complete an anonymous online questionnaire. No financial incentives were provided for participation. Because recruitment was conducted through participating community settings, the exact number of individuals who received an invitation and characteristics of non-respondents were not available. Data were collected using questionnaires administered across several workplaces and educational settings in Northeast Iowa. Prior to participation, all respondents provided informed consent after receiving information regarding the aims of the study, confidentiality, voluntariness of participation, and the right to withdraw at any time without penalty. The study protocol was approved by the Luther College Human Subjects Review Board (HSRB) and was conducted in accordance with institutional ethical standards.

Initially, 569 individuals participated in the study. Cases with substantial missing data (i.e., questionnaires containing less than 20% completed responses across the study measures) were excluded from further analyses because they did not provide sufficient information for reliable profile estimation and could bias latent profile classification. This threshold was used to remove highly incomplete survey submissions while retaining participants who provided sufficient information across the study measures for reliable estimation and comparison. Participants with adequately completed questionnaires were retained, resulting in a final analytic sample of 522 individuals included in the LPA. After these exclusions, remaining item-level missingness was limited; therefore, analyses were conducted using complete available data for the variables included in each model, rather than applying additional missing-data correction procedures. Inspection of missingness across instruments did not indicate disproportionate missing data for any specific questionnaire or domain, reducing concern that instrument-specific missingness systematically influenced the profile solution or subsequent comparisons.

Participants ranged in age from 18 to 70 years (*M* = 39.74, *SD* = 13.27). The average height was 66.10 inches (*SD* = 4.07), the average weight was 170.17 pounds (*SD* = 39.62), and the mean body mass index (BMI) was 27.38 (*SD* = 5.90). Women constituted 80.3% of the sample, whereas men represented 19.7% (see Table 1). Most participants identified as White/non-Hispanic (99.2%). Regarding marital status, 79.3% were married, 10.5% had never married, 8.5% were divorced, 1.3% were widowed, and 0.4% were separated. In terms of educational attainment, the largest proportion reported having an associate degree (34.6%), followed by some college education without a degree (21.9%), a high school diploma or GED (17.9%), a bachelor’s degree (12.7%), and a master’s degree (7.7%). Smaller proportions reported professional or doctoral degrees.

### 2.2. Procedure

Participants completed an online survey administered via the Qualtrics platform. The procedure involved completing a battery of standardized self-report questionnaires assessing dietary and lifestyle behaviors, perceived stress, depressive symptoms, coping self-efficacy, daily spiritual experiences, religious/spiritual meaning, values, and beliefs, religiousness/spirituality, gratitude, and forgiveness. Participants responded individually and anonymously, and no identifying information was linked to survey responses. Survey completion required approximately 30 min on average.

### 2.3. Measures

#### 2.3.1. Perceived Stress

Perceived stress was assessed using the 10-item Perceived Stress Scale (PSS-10) developed by Cohen et al. [45]. The scale measures the degree to which individuals perceive situations in their lives as unpredictable, uncontrollable, and overwhelming during the previous month. Sample items include: “In the last month, how often have you felt nervous and stressed?” and “In the last month, how often have you felt difficulties were piling up so high that you could not overcome them?” Responses were rated on a 5-point scale ranging from 0 (never) to 4 (very often). Positively worded items were reverse-scored, with higher scores indicating greater perceived stress. In the present study, Cronbach’s α for the scale was 0.86.

#### 2.3.2. Depressive Symptoms

Depressive symptoms were assessed using the 10-item version of the Center for Epidemiologic Studies Depression Scale (CES-D-10) developed by Radloff [46]. The scale measures the frequency of depressive symptoms experienced during the previous week. Sample items include: “I felt depressed” and “I felt lonely.” Participants responded using a 4-point scale ranging from 0 (rarely or none of the time) to 3 (all of the time). Positively worded items were reverse scored, with higher scores indicating greater depressive symptom severity. In the present study, Cronbach’s α for the scale was 0.86.

#### 2.3.3. Coping Self-Efficacy

Coping self-efficacy was assessed using the Coping Self-Efficacy Scale developed by Chesney et al. [47]. The scale consists of 26 items assessing individuals’ confidence in their ability to cope effectively with stress and adversity. The measure comprises three aspects of coping self-efficacy, namely problem-focused coping, stopping unpleasant emotions and thoughts, and obtaining support from family and friends, which together form a higher-order coping self-efficacy factor. Participants responded to the stem: “When things aren’t going well for you, or when you’re having problems, how confident or certain are you that you can do the following.” Sample items include: “Sort out what can be changed and what cannot be changed” and “Get emotional support from friends or family.” Responses ranged from 0 (cannot do at all) to 10 (certain can do), with higher scores indicating greater coping self-efficacy. In the present study, Cronbach’s α for the total scale was 0.92.

#### 2.3.4. Daily Spiritual Experiences

Daily spiritual experiences were assessed using the 6-item version of the Daily Spiritual Experience Scale (DSES) developed by Underwood and Teresi [48]. The scale measures the frequency of ordinary spiritual experiences in everyday life, including inner peace, connectedness, and transcendent experiences. Sample items include: “I feel deep inner peace or harmony” and “I am spiritually touched by the beauty of creation.” Responses were rated on a 6-point scale ranging from 1 (never) to 6 (many times a day), with higher scores indicating more frequent daily spiritual experiences. Item scores were averaged to create an overall index of daily spiritual experiences. In the present study, Cronbach’s α for the scale was 0.91.

#### 2.3.5. Religious/Spiritual Meaning, Values, and Beliefs

Religious/spiritual meaning, values, and beliefs were assessed using four items forming the Meaning and Values subscales of the Brief Multidimensional Measure of Religiousness/Spirituality (BMMRS) [49]. The BMMRS is a widely used multidimensional instrument for assessing religiousness and spirituality in health and behavioral research, and its domains align with common conceptualizations of religious/spiritual functioning. These items assessed beliefs concerning meaning and purpose in life, divine guidance, moral responsibility, and worldview. Sample items included: “The events in my life unfold according to a divine or greater plan” and “I have a sense of mission or calling in my own life.” Responses were rated on a 4-point scale ranging from 1 (strongly disagree) to 4 (strongly agree), with higher scores indicating stronger meaning, values, and beliefs orientations. In the present study, Cronbach’s α for the scale was 0.78.

#### 2.3.6. Religiousness and Spirituality

Religiousness and spirituality were assessed using the Overall Self-Ranking subscale of the BMMRS [49]. Participants were asked: “To what extent do you consider yourself a religious person?” and “To what extent do you consider yourself a spiritual person?” Responses were rated on a 4-point scale ranging from 1 (not at all) to 4 (a great deal), with higher scores indicating greater self-perceived religiousness and spirituality. Because the two items were strongly conceptually related and highly correlated (*r* = 0.78, *p* < 0.001), they were combined into a single composite indicator reflecting overall self-perceived religiousness/spirituality.

#### 2.3.7. Gratitude

Dispositional gratitude was measured using the Gratitude Questionnaire–Six Item Form (GQ-6) developed by McCullough et al. [50]. The GQ-6 is a unidimensional measure assessing the tendency to experience gratitude in everyday life. Sample items include: “I have so much in life to be thankful for” and “I am grateful to a wide variety of people.” Participants responded using a scale ranging from 1 (strongly disagree) to 7 (strongly agree), with higher scores indicating greater dispositional gratitude. In the present study, Cronbach’s α for the scale was 0.85.

#### 2.3.8. Forgiveness

Dispositional forgiveness was assessed using the Tendency to Forgive Scale (TTF) developed by Brown [51]. The TTF is a four-item measure assessing individuals’ general tendency to forgive interpersonal transgressions. Sample items include: “I tend to get over it quickly when someone hurts my feelings” and “I have a tendency to harbor grudges” (reverse scored). Participants responded using a scale ranging from 1 (strongly disagree) to 7 (strongly agree), with higher scores indicating a greater tendency to forgive. In the present study, Cronbach’s α for the scale was 0.74.

#### 2.3.9. Dietary and Lifestyle Behaviors

Dietary and lifestyle behaviors were assessed using a modified version of the Nutrition Questionnaire About Eating Practices derived from the Personal Wellness Profile developed by the Parklawn Federal Occupational Health Unit [52]. The questionnaire consisted of 22 single-item behavioral indicators covering a broad range of eating practices and health-related behaviors, including breakfast consumption, snack and fast-food intake, food choices, fruit and vegetable consumption, salt use, water intake, supplementation, dieting behavior, caffeine intake, alcohol use, and smoking (see Appendix A). Given the multidimensional and behavioral nature of the questionnaire, individual indicators were analyzed separately in the LPA to provide a more detailed representation of dietary and lifestyle patterns. Additionally, for descriptive purposes and bivariate associations reported in Appendix B, a general nutrition index was calculated by averaging standardized item scores after reverse coding negatively oriented items, with higher scores indicating healthier dietary and lifestyle behaviors. Because the questionnaire assessed relatively heterogeneous behavioral domains, the composite index should be interpreted cautiously despite acceptable internal consistency (α = 0.71).

### 2.4. Statistical Analysis

In the initial step, preliminary analyses including data screening, descriptive statistics, reliability analyses, and bivariate correlations were conducted for all study variables. Inspection of skewness and kurtosis values indicated that the distributions of the study variables did not substantially deviate from normality, and Levene’s tests supported the assumption of homogeneity of variance for the conducted analyses of variance (ANOVAs). LPA was performed to identify subgroups of participants characterized by distinct patterns of psychological functioning. The profile indicators included perceived stress, depressive symptoms, coping self-efficacy, daily spiritual experiences, religious/spiritual meaning, values, and beliefs, religiousness/spirituality, gratitude, and forgiveness. Models specifying one through seven latent classes were estimated and compared. The following criteria were used to determine the optimal profile solution: Akaike Information Criterion (AIC), Bayesian Information Criterion (BIC), sample size-adjusted Bayesian Information Criterion (SABIC), and entropy values. Lower AIC, BIC, and SABIC values indicated better model fit, whereas higher entropy values reflected better classification accuracy [53]. The final model selection also considered parsimony, interpretability, and profile size adequacy. After identifying the optimal latent profile solution, analyses of variance (ANOVAs) were conducted to examine differences between profiles in psychological, dietary, and lifestyle variables. Post hoc comparisons were performed using Tukey’s honestly significant difference (HSD) test. Additionally, analyses of covariance (ANCOVAs) were conducted to examine differences in dietary and lifestyle behaviors across latent profiles while controlling for age. Partial eta squared (η^2^p) was reported as a measure of effect size for ANOVA and ANCOVA results. Statistical significance was established at *p* < 0.05. All analyses were conducted using Jamovi (Version 2.4.7; The Jamovi Project, Sydney, Australia).

## 3. Results

Descriptive statistics, skewness, kurtosis, and bivariate correlations are presented in Appendix B. For correlational analyses, the nutrition variable was operationalized as a composite index reflecting overall dietary and lifestyle behaviors. The nutrition index demonstrated small but significant negative correlations with perceived stress and depressive symptoms, and positive correlations with coping self-efficacy, gratitude, and forgiveness, whereas associations with daily spiritual experiences, religious/spiritual meaning, values, and beliefs, and religiousness/spirituality were non-significant. Additionally, the nutrition index showed a small positive correlation with educational attainment (*r* = 0.11, *p* = 0.025), based on available data for this analysis.

After preliminary data screening, LPA was conducted to identify distinct subgroups of participants characterized by different psychological patterns. Models specifying one through seven classes were estimated to ensure a comprehensive evaluation of possible solutions. Although AIC values decreased with increasing numbers of classes, both BIC and SABIC reached their lowest values for the four-class model. In addition, this solution demonstrated the highest entropy (0.852), indicating good classification accuracy (Table 2). Based on these criteria, the four-class solution was selected as the optimal and most parsimonious representation of the data.

**Figure 1:**
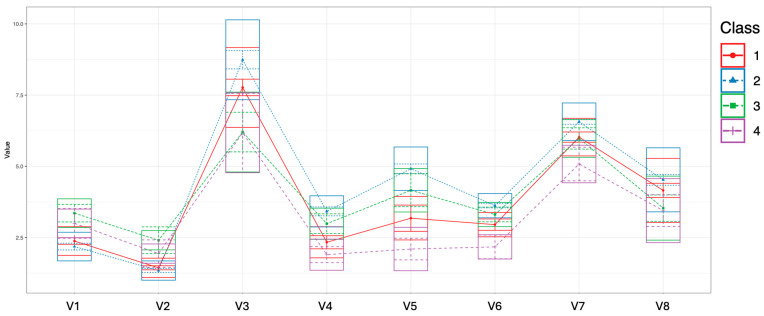
Latent psychospiritual profiles across psychological and spiritual indicators (V1 = Perceived stress, V2 = Depressive symptoms, V3 = Coping self-efficacy, V4 = Religiousness and spirituality, V5 = Daily spiritual experiences, V6 = R/S meaning/values/beliefs, V7 = Gratitude, V8 = Forgiveness).

The four identified profiles differed meaningfully across psychological variables (Figure 1; Table 3). Profile numbering reflects the order generated by the LPA output and should not be interpreted as an a priori ordered typology. Class 1 (Moderate; *n* = 195) was characterized by intermediate levels of perceived stress and generally favorable psychological functioning, including relatively low depressive symptoms and moderate levels of coping self-efficacy, religious/spiritual functioning, gratitude, and forgiveness. Class 2 (Flourishing; *n* = 199) reflected the most adaptive profile, with the lowest levels of perceived stress together with similarly low depressive symptoms and the highest levels across all examined psychological and spiritual resources. Class 3 (Vulnerable; *n* = 70) demonstrated the highest levels of perceived stress and depressive symptoms, accompanied by relatively low coping self-efficacy and forgiveness, but comparatively elevated religiousness/spirituality, daily spiritual experiences, and religious/spiritual meaning, values, and beliefs. In contrast, Class 4 (Maladaptive; *n* = 58) was characterized by consistently the lowest levels across nearly all positive psychological and spiritual resources together with elevated perceived stress and depressive symptoms, suggesting limited coping capacities.

Next, differences across profiles were examined for dietary and lifestyle behaviors (Table 4). Significant effects were observed for several indicators. Participants in the Maladaptive profile reported more frequent breakfast skipping compared to those in the Moderate and Flourishing profiles. Lower frequency of fast-food consumption was observed in the Moderate and Flourishing profiles relative to the Maladaptive profile, while whole-grain consumption was higher in the Flourishing profile compared to the Maladaptive profile. Additionally, both the Vulnerable and Maladaptive profiles reported less frequent consumption of leafy green vegetables compared to the Flourishing profile. Further differences were found for sweets and dessert consumption, with higher levels in the Vulnerable and Moderate profiles compared to the Flourishing profile. Salt intake was higher in the Moderate and Maladaptive profiles relative to the Flourishing profile. Moreover, absence of calcium supplementation was more common in the Moderate and Maladaptive profiles compared to the Flourishing profile. No significant differences were observed for snacking frequency, fat intake type, protein source, grain intake, fruit and dairy consumption, total fat intake, water intake, long-term weight change, dieting behavior, caffeine consumption, alcohol use, or smoking (*p*s > 0.05). Because age differed significantly across latent profiles, additional analyses of covariance (ANCOVAs) controlling for age were conducted and are presented in Appendix C. No significant differences were found for BMI.

## 4. Discussion

The present study investigated whether distinct psychospiritual profiles are associated with dietary and lifestyle behaviors using a person-centered approach. Although no a priori assumptions were made regarding the number or specific structure of latent profiles, it was expected that profiles characterized by more adaptive psychospiritual functioning would demonstrate relatively healthier dietary and lifestyle behaviors. Consistent with these expectations, four distinct profiles were identified, namely Moderate, Flourishing, Vulnerable, and Maladaptive, differing substantially in levels of perceived stress, depressive symptoms, coping self-efficacy, gratitude, forgiveness, and dimensions of religiousness and spirituality. Importantly, these profiles also differed across several dietary indicators, suggesting associations between psychospiritual functioning and eating-related behaviors. Moreover, by incorporating multiple dimensions of both psychological distress and positive psychospiritual functioning, the present study allowed for a potentially more differentiated representation of individual functioning than simpler low–moderate–high flourishing classifications based on narrower sets of variables [54].

Among the identified profiles, the Flourishing profile demonstrated the most adaptive overall pattern of functioning, characterized by low perceived stress and depressive symptoms together with high levels of coping self-efficacy, gratitude, forgiveness, daily spiritual experiences, religious/spiritual meaning and beliefs, and self-perceived religiousness/spirituality. This profile was additionally associated with more favorable dietary behaviors, including lower breakfast skipping and fast-food consumption, greater whole-grain and vegetable intake, lower salt use, lower intake of sweets and desserts, and more frequent calcium supplementation. Although the Moderate profile demonstrated less favorable psychospiritual functioning relative to the Flourishing group, it was generally characterized by more adaptive overall functioning than the Vulnerable and Maladaptive profiles, suggesting a gradient-like relationship between overall psychospiritual functioning and health-related behaviors.

These findings extend previous variable-centered research [10,18,30,33] by suggesting that healthier dietary patterns may be linked not only to isolated psychological characteristics, but also to broader constellations of emotional, coping-related, and spiritual resources co-occurring within individuals. The findings are additionally consistent with broaden-and-build perspectives [29], which suggest that positive emotional functioning may support more flexible self-regulation and health-related decision making over time, whereas relational spirituality frameworks emphasize the role of meaning-related and interpersonal resources in adaptive functioning and coping [43].

The Vulnerable and Maladaptive profiles provided important insight into the heterogeneity of psychospiritual functioning. Notably, the Vulnerable profile demonstrated the highest levels of perceived stress and depressive symptoms despite relatively elevated religious and spiritual resources. In contrast, the Maladaptive profile was characterized by consistently low levels of nearly all positive psychological and spiritual resources together with elevated perceived stress and depressive symptoms. These patterns may tentatively suggest that higher levels of religiousness and spirituality do not necessarily preclude psychological distress. Rather, they may sometimes become particularly salient during periods of distress and reflect attempts to cope with adversity rather than uniformly adaptive functioning [55,56]. In this context, some forms of institutionalized religious involvement have occasionally been interpreted as potential markers of elevated stress or psychological burden [57]. Supplementary correlational analyses further suggested that general nutrition scores were not significantly associated with religiousness/spirituality-related variables when examined independently, unlike most of the remaining psychological variables.

Alternatively, it is possible that more adaptive psychospiritual functioning is associated not merely with elevated spirituality itself, but with spirituality embedded within broader patterns of positive emotional, interpersonal, and self-related resources, broadly consistent with a relational perspective on spirituality [43,58]. Moreover, the distinction between the Vulnerable and Maladaptive profiles may also be consistent with dual-continua perspectives on mental health, which propose that psychological distress and positive functioning represent related but partially independent dimensions rather than opposite ends of a single continuum [21,22,59].

The less adaptive profiles were additionally characterized by less favorable dietary patterns. These findings are broadly consistent not only with previous psychological literature linking elevated distress and lower coping resources with poorer dietary choices [10,13,15], but also correspond with the reward-based stress eating model, which proposes that chronic stress may increase the motivational salience and rewarding value of highly palatable foods through interactions between neuroendocrine stress responses and reward-related processes [16]. From this perspective, maladaptive eating behaviors may partly serve short-term regulatory or self-soothing functions under conditions of psychological burden despite potentially adverse long-term health consequences. Such interpretations are additionally consistent with broader literature emphasizing the role of emotion regulation difficulties, interpersonal distress, and maladaptive coping processes in unhealthy eating-related behaviors and associated psychological difficulties [60].

The observed associations appeared selective rather than global, as psychospiritual profiles differentiated only some dietary and lifestyle indicators, and the observed effect sizes were generally small. This pattern is broadly consistent with previous literature suggesting that eating-related behaviors are influenced by multiple interacting psychological and contextual factors, while individual psychological resources are often associated with specific rather than universal aspects of dietary functioning [10,18]. Previous findings indicate that coping-related and emotional-regulation processes may be especially relevant to emotionally salient eating-related behaviors, including stress eating, irregular eating patterns, and consumption of highly palatable foods [13,15,16]. Although the observed effects were modest, they may nevertheless remain clinically and socially meaningful given that the examined dietary tendencies are consistently associated with obesity, cardiovascular disease, atherosclerosis, metabolic disorders, and other major chronic health conditions [61,62]. Moreover, at least some of the examined psychological resources appear modifiable through relatively low-cost and scalable psychological or behavioral interventions targeting stress regulation, gratitude, forgiveness, coping-related capacities, and meaning-focused functioning [63,64]. Consequently, even modest improvements in psychospiritual functioning could potentially contribute not only to better psychological well-being, but also to more adaptive dietary and lifestyle tendencies at the population level.

Additional analyses controlling for age indicated that most differences between profiles remained significant even after adjusting for age-related variance, with only the effects for whole-grain consumption and daily vegetable servings becoming non-significant, suggesting that the observed associations between psychospiritual functioning and dietary behaviors were not reducible solely to age differences. Moreover, age itself was associated with several dietary and lifestyle indicators, including less frequent leafy green vegetable consumption, greater daily grain and dairy consumption, lower sweets and dessert intake, and more frequent calcium supplementation. These findings are broadly consistent with previous literature, suggesting that older adults may engage more frequently in selected health-promoting behaviors, potentially due to greater health awareness, medical recommendations, or increasing concerns regarding chronic disease prevention [65,66]. Interestingly, age was also positively associated with caffeine consumption. This finding may partly reflect generational differences in preferred stimulant use, as younger individuals increasingly consume alternative products such as energy drinks, which may not have been fully captured by the present measure [67,68]. Furthermore, the finding that the Flourishing profile was characterized by significantly older age relative to the remaining profiles is broadly consistent with evidence suggesting that older adults often report higher well-being and may regulate emotional experiences more effectively across adulthood [69]. However, this advantage may not extend indefinitely, as very advanced age may involve cumulative health-related limitations that negatively affect well-being and functioning [70].

### 4.1. Practical Implications

The present findings may carry several practical implications for health promotion and preventive interventions. The identified profiles suggest that dietary and lifestyle behaviors may be associated not only with isolated psychological characteristics, but also with broader configurations of emotional, coping-related, and spiritual functioning. Consequently, interventions supporting stress regulation, coping self-efficacy, gratitude, forgiveness, and meaning-focused functioning may potentially contribute not only to psychological well-being, but also to selected health-related behaviors. Such approaches may include relatively low-cost and scalable strategies, for example, gratitude journaling or writing exercises, structured self-forgiveness workbooks, or meaning-centered therapeutic techniques focused on values clarification and purpose-oriented reflection [63,64,71]. Together with previous literature [37,55,56], the present findings additionally suggest that clinicians and health professionals may benefit from considering the multidimensional and heterogeneous nature of religiousness and spirituality within broader psychosocial assessment, as these factors may function as either adaptive resources or indicators of psychological struggle depending on the context in which they are experienced and expressed.

### 4.2. Limitations

Several limitations of the present study should be acknowledged. First, the cross-sectional design precludes causal inferences regarding the relationships between psychospiritual functioning and dietary or lifestyle behaviors. Although the present findings are broadly consistent with theoretical perspectives suggesting that psychospiritual resources may support adaptive health-related functioning, previous literature also indicates that these relationships are likely bidirectional, as dietary behaviors may themselves influence emotional functioning, stress regulation, and well-being over time [1,2]. Second, the study did not assess religious affiliation or participants’ formal religious status. Although the employed measures may be administered across individuals with varying levels of religiosity or spirituality, including nonreligious participants who may endorse lower response options, the meaning and psychological function of religious/spiritual constructs may differ substantially across religious, spiritual, and nonreligious groups, as well as across different religious traditions. Consequently, the observed profiles and associations may not generalize equally across all belief systems or cultural contexts. Nevertheless, it should be noted that the sample was recruited in the United States, where Christianity remains the majority religious affiliation, with recent national estimates indicating that approximately two-thirds of adults identify as Christian [72]. Furthermore, although the present study included multiple dimensions of religiousness and spirituality, incorporating measures reflecting more instrumental or potentially maladaptive forms of spiritual functioning, such as spiritual bypassing [56], might provide additional insight into the heterogeneity of psychospiritual functioning. Third, the study relied on a convenience sample and was predominantly composed of women. Although the number of male participants remained sufficiently large to retain men within the analyses, the obtained profiles and associations should nevertheless be generalized with caution, particularly to more gender-balanced or culturally diverse populations. Fourth, dietary and lifestyle behaviors were compared across latent profiles using assigned profile membership as an observed grouping variable. Although this approach provides an interpretable first examination of behavioral differences across profiles, it does not fully account for classification uncertainty. Future studies could strengthen these analyses by using distal outcome procedures specifically designed for mixture models, such as the Bolck–Croon–Hagenaars distal outcome approach. Fifth, the latent profile solution should be interpreted as exploratory and model-generating rather than confirmatory. Although the four-profile structure was supported in the present sample, its stability requires evaluation in independent datasets, as alternative profile solutions may provide a better fit in other populations. Finally, the present findings were obtained in a general community sample. Psychospiritual resources and their associations with dietary and lifestyle behaviors may operate differently under conditions of severe psychological crisis, trauma, chronic illness, or other major stressors.

## 5. Conclusions

The present study preliminarily demonstrated that distinct psychospiritual profiles are associated with modest differences in selected dietary and lifestyle behaviors, particularly breakfast consumption, fast-food intake, vegetable and whole-grain consumption, sweets and dessert intake, salt use, and calcium supplementation. Using a person-centered approach, the findings suggest that healthier dietary tendencies may be linked not only to lower psychological distress, but also to broader constellations of coping-related, emotional, interpersonal, and spiritual resources co-occurring within individuals. At the same time, the identified profiles highlighted the heterogeneous nature of religiousness and spirituality, indicating that elevated spiritual functioning does not necessarily preclude psychological burden and may reflect different psychological functions depending on the broader psychosocial context. Collectively, the findings support the value of integrating psychological and spiritual dimensions within research on health-related behaviors and suggest that psychospiritual functioning may represent one component of broader processes associated with adaptive lifestyle patterns.

## Figures and Tables

**Table 1:** Sociodemographic characteristics of the study participants.

Variable	*n*	%
Sex		
Female	419	80.3
Male	103	19.7
Ethnicity		
White/non-Hispanic	518	99.2
Black/African American	1	0.2
Asian/Hispanic/Other	3	0.6
Marital status		
Married	414	79.3
Widowed	7	1.3
Divorced	44	8.5
Separated	2	0.4
Never married	55	10.5
Education		
No schooling completed	4	0.8
High school diploma/GED	94	17.9
Some college, no degree	114	21.9
Associate degree	181	34.6
Bachelor’s degree	66	12.7
Master’s degree	40	7.7
Professional degree	3	0.6
Doctorate degree	20	3.8

**Table 2:** Model Fit Indices for Latent Profile Solutions.

Classes	LogLik	AIC	BIC	SABIC	Entropy
1	−5291	10,614	10,682	10,632	1
2	−4899	9849	9955	9876	0.829
3	−4752	9572	9717	9609	0.838
4	−4651	9387	9570	9434	0.852
5	−4625	9385	9607	9443	0.845
6	−4608	9392	9653	9460	0.832
7	−4595	9404	9704	9482	0.821

**Table 3:** Psychological Profiles Across Latent Profiles.

Variable	Class 1 (Moderate) *M* (*SE*) (*n* = 195)	Class 2 (Flourishing) *M* (*SE*) (*n* = 199)	Class 3 (Vulnerable) *M* (*SE*) (*n* = 70)	Class 4 (Maladaptive) *M* (*SE*) (*n* = 58)	*F*_(3, 518)_	*p*	Partial η^2^	Post Hoc
Perceived stress	2.38 (0.06)	2.18 (0.06)	3.36 (0.15)	3.00 (0.24)	115.7	<0.001	0.40	3 > 4 > 1 > 2
Depressive symptoms	1.44 (0.04)	1.33 (0.03)	2.44 (0.07)	1.96 (0.06)	102.6	<0.001	0.37	3 > 4 > 1 ≈ 2
Coping self-efficacy	7.77 (0.14)	8.74 (0.14)	6.20 (0.34)	6.18 (0.62)	78.9	<0.001	0.31	2 > 1 > 3 ≈ 4
Daily spiritual experiences	3.18 (0.23)	4.92 (0.09)	4.16 (0.29)	2.10 (0.24)	333.4	<0.001	0.66	2 > 3 > 1 > 4
R/S meaning/values/beliefs	2.95 (0.10)	3.62 (0.04)	3.31 (0.13)	2.17 (0.25)	160.5	<0.001	0.48	2 > 3 > 1 > 4
Religiousness and spirituality	2.33 (0.11)	3.42 (0.08)	2.99 (0.18)	1.90 (0.16)	195.6	<0.001	0.53	2 > 3 > 1 > 4
Gratitude	6.03 (0.10)	6.56 (0.04)	5.98 (0.18)	5.08 (0.30)	61.4	<0.001	0.26	2 > 1 ≈ 3 > 4
Forgiveness	4.15 (0.10)	4.53 (0.09)	3.53 (0.24)	3.45 (0.23)	18.4	<0.001	0.10	2 > 1 > 3 ≈ 4

Note. Post hoc comparisons were conducted using Tukey’s HSD test; R/S = religious/spiritual.

**Table 4:** Differences in Dietary and Lifestyle Behaviors Across Latent Profiles.

Variable	Class 1 (Moderate) *M* (*SD*) (*n* = 195)	Class 2 (Flourishing) *M* (*SD*) (*n* = 199)	Class 3 (Vulnerable) *M* (*SD*) (*n* = 70)	Class 4 (Maladaptive) *M* (*SD*) (*n* = 58)	*F*_(3, 518)_	*p*	Partial η^2^	Post Hoc
Breakfast-skipping frequency	2.22 (1.16)	2.05 (1.19)	2.33 (1.23)	2.73 (1.18)	4.48	0.005	0.03	4 > 2; 4 > 1
Low frequency of snack food consumption between meals	2.64 (0.82)	2.70 (0.82)	2.38 (0.96)	2.52 (0.77)	2.14	0.098	0.01	ns
Low frequency of fast-food consumption	3.03 (0.72)	3.16 (0.74)	2.98 (0.62)	2.69 (0.85)	4.58	0.004	0.03	1 > 4; 2 > 4
Low-fat food consumption	3.34 (0.71)	3.46 (0.77)	3.23 (0.89)	3.15 (0.88)	2.63	0.052	0.02	ns
Whole-grain consumption	3.15 (1.00)	3.36 (0.98)	3.05 (1.02)	3.00 (1.13)	2.92	0.036	0.02	2 > 4
Plant-based protein consumption	2.03 (0.81)	2.12 (0.83)	2.03 (0.99)	2.06 (1.10)	0.49	0.688	<0.01	ns
Low frequency of leafy green vegetable intake	2.51 (1.12)	2.30 (1.04)	2.72 (1.12)	2.79 (1.20)	4.12	0.008	0.02	3 > 2; 4 > 2
Daily grain servings	2.71 (1.02)	2.58 (0.89)	2.81 (1.02)	2.66 (0.78)	1.13	0.338	0.01	ns
Daily vegetable servings	2.91 (0.92)	3.09 (1.05)	2.83 (0.79)	2.71 (0.90)	2.93	0.035	0.02	2 > 4
Daily fruit servings	2.76 (0.91)	2.93 (1.07)	2.77 (1.06)	2.63 (1.04)	1.58	0.197	0.01	ns
Daily dairy servings	3.39 (1.10)	3.30 (1.12)	3.52 (1.23)	3.06 (1.25)	1.39	0.247	0.01	ns
Daily protein servings	3.15 (1.00)	2.97 (0.89)	3.10 (0.92)	3.02 (1.21)	1.31	0.275	0.01	ns
Daily sweets and dessert servings	2.66 (1.09)	2.37 (0.90)	2.95 (1.27)	2.71 (1.01)	5.85	<0.001	0.03	3 > 2; 1 > 2
Daily fat servings	2.58 (0.88)	2.45 (0.81)	2.70 (1.00)	2.44 (0.99)	1.70	0.171	0.01	ns
Frequency of salt addition or salty food consumption	1.98 (0.90)	1.76 (0.77)	1.97 (0.82)	2.15 (0.85)	4.31	0.006	0.02	1 > 2; 4 > 2
Daily water intake (glasses)	4.23 (2.20)	4.29 (2.23)	3.95 (2.30)	4.04 (2.45)	0.42	0.736	<0.01	ns
Current weight relative to 10 years ago (lower weight)	2.17 (0.94)	2.24 (1.04)	2.12 (0.85)	2.25 (0.98)	0.38	0.765	<0.01	ns
Calcium supplementation (no use)	1.76 (0.43)	1.61 (0.49)	1.70 (0.46)	1.83 (0.38)	5.59	0.001	0.03	1 > 2; 4 > 2
Absence of dieting behavior	1.73 (0.45)	1.71 (0.45)	1.60 (0.49)	1.65 (0.48)	1.38	0.251	0.01	ns
Daily caffeine intake	2.81 (1.43)	2.93 (1.53)	3.35 (1.76)	2.83 (1.46)	1.62	0.187	0.01	ns
Alcohol consumption	1.63 (0.70)	1.55 (0.73)	1.45 (0.68)	1.79 (1.05)	1.85	0.140	0.01	ns
Smoking behavior	1.46 (0.94)	1.44 (0.92)	1.38 (0.87)	1.85 (1.30)	1.63	0.186	0.01	ns
Age	36.42 (12.56)	44.77 (12.90)	36.45 (11.58)	35.98 (13.28)	18.11	<0.001	0.09	2 > 1, 3, 4
Body mass index (BMI)	27.24 (5.82)	27.31 (5.80)	27.43 (6.26)	27.26 (5.33)	0.02	0.997	<0.01	ns

Note. Post hoc comparisons were conducted using Tukey’s HSD test; ns = non-significant.

## Data Availability

The dataset underpinning the conclusions of this research is accessible upon request from the corresponding author, S.B.S.-B. These data are not publicly available to protect the confidentiality of research participants and prevent any potential compromise of their privacy.

## Abbreviations

The following abbreviations are used in this manuscript:

ANCOVA	Analysis of Covariance
ANOVA	Analysis of Variance
AIC	Akaike Information Criterion
BIC	Bayesian Information Criterion
BMI	Body Mass Index
BMMRS	Brief Multidimensional Measure of Religiousness/Spirituality
CES-D-10	10-item Center for Epidemiologic Studies Depression Scale
DSES	Daily Spiritual Experience Scale
GED	General Educational Development
GQ-6	Gratitude Questionnaire–Six Item Form
HSD	Honestly Significant Difference
HSRB	Human Subjects Review Board
LPA	Latent Profile Analysis
PSS-10	10-item Perceived Stress Scale
R/S	Religious/Spiritual
SABIC	Sample-Adjusted Bayesian Information Criterion
SD	Standard Deviation
SE	Standard Error
TTF	Tendency to Forgive Scale

## Appendix A. Dietary and Health Behavior Items Adapted from the Personal Wellness Profile (Parklawn Federal Occupational Health Unit, 2000)

1. How often do you eat breakfast (more than just a roll and a cup of coffee)? (from 1 = Eat breakfast every day to 4 = Seldom or never eat breakfast)
2. How often do you eat snack foods between meals (e.g., chips, pastries, soft drinks, candy, ice cream, cookies)? (from 1 = Three or more times per day to 4 = Seldom or never eat snack foods)
3. How often do you eat fast food meals (e.g., hamburgers, tacos, fried chicken, hot dogs, French fries, milkshakes)? (from 1 = Four or more times per week to 4 = Seldom or never)
4. Indicate the kinds of foods you usually eat. (from 1 = Nearly always eat high-fat foods to 5 = Eat only low-fat foods)
5. Indicate the kinds of breads and grains you usually eat. (from 1 = Nearly always eat refined grain products to 5 = Eat only whole-grain products)
6. Indicate the kinds of protein foods you usually eat. (from 1 = Nearly always eat animal-based proteins to 5 = Eat only plant-based proteins)
7. How often do you eat dark leafy green vegetables (e.g., spinach, kale, broccoli)? (from 1 = Five or more times per week to 5 = Seldom or never)
8. How many servings of grains do you typically consume per day? (from 0 = 0 servings to 5 = 5 or more servings)
9. How many servings of vegetables do you typically consume per day? (from 0 = 0 servings to 5 = 5 or more servings)
10. How many servings of fruit do you typically consume per day? (from 0 = 0 servings to 5 = 5 or more servings)
11. How many servings of dairy products do you typically consume per day? (from 0 = 0 servings to 5 = 5 or more servings)
12. How many servings of protein foods do you typically consume per day? (from 0 = 0 servings to 5 = 5 or more servings)
13. How many servings of sweets and desserts do you typically consume per day? (from 0 = 0 servings to 5 = 5 or more servings)
14. How many servings of fats (e.g., butter, oil, margarine, dressings) do you typically consume per day? (from 0 = 0 servings to 5 = 5 or more servings)
15. How often do you add salt to your food or consume salty foods (e.g., chips, pickles, soy sauce)? (from 1 = Seldom or never to 4 = Nearly every meal)
16. How many 8-ounce glasses of water do you usually drink each day? (from 1 = One or less to 8 = Eight or more)
17. Compared to your weight 10 years ago, how much do you weigh now? (from 1 = 10 pounds more to 4 = Weigh less now)
18. Do you take a calcium supplement regularly? (from 1 = Yes to 2 = No)
19. Do you diet often (at least 1–2 times per year)? (from 1 = Yes to 2 = No)
20. How many caffeinated drinks do you usually consume per day (e.g., coffee, tea, cola)? (from 0 = None to 6 = Six or more per day)
21. How many alcoholic drinks do you usually consume in a typical week? (one drink = one beer, one glass of wine, one shot of liquor, or one mixed drink) (from 1 = Seldom or never to 5 = 21 or more drinks per week)
22. Which of the following best describes your smoking status? (from 1 = Never smoked to 5 = Currently smoke more than 10 cigarettes per day)

## Appendix B

**Table A1:** Descriptive Statistics and Intercorrelations Among Study Variables (*N* = 522).

#	*M* (*SD*)	Skewness	Kurtosis	1	2	3	4	5	6	7	8
1	2.47 (0.64)	0.33	0.09	—							
2	1.56 (0.49)	1.59	3.19	0.67 ***	—						
3	7.83 (1.70)	−0.4	0.03	−0.54 ***	−0.51 ***	—					
4	3.88 (1.23)	−0.25	−0.64	−0.19 ***	−0.14 **	0.30 ***	—				
5	3.18 (0.61)	−0.85	1.1	−0.14 **	−0.13 **	0.29 ***	0.70 ***	—			
6	2.80 (0.79)	−0.02	−0.75	−0.13 **	−0.10 *	0.23 ***	0.77 ***	0.61 ***	—		
7	6.15 (0.79)	−1.09	1.01	−0.25 ***	−0.28 ***	0.39 ***	0.44 ***	0.45 ***	0.36 ***	—	
8	4.16 (1.19)	−0.06	−0.3	−0.37 ***	−0.27 ***	0.39 ***	0.17 ***	0.16 ***	0.15 **	0.18 ***	—
9	2.93 (0.26)	−0.04	−0.13	−0.16 ***	−0.11 *	0.16 ***	0.01	0.03	0.03	0.16 ***	0.17 ***

Note. 1 = Perceived stress; 2 = Depressive symptoms; 3 = Coping self-efficacy; 4 = Daily spiritual experiences; 5 = Religious/spiritual meaning/values/beliefs; 6 = Religiousness and spirituality; 7 = Gratitude; 8 = Forgiveness; 9 = Nutrition; * *p* < 0.05, ** *p* < 0.01, *** *p* < 0.001.

## Appendix C

**Table A2:** ANCOVA Effects for Dietary and Lifestyle Behaviors Across Latent Profiles Controlling for Age.

Variable	Profile Membership *F*_(3, 385)_	Profile Membership *p*	Age *F*_(1, 385)_	Partial η^2^ Profile	Age *p*	Partial η^2^ Age
Breakfast-skipping frequency	3.07	0.028	0.02	0.72	0.398	<0.01
Low frequency of snack food consumption between meals	2.38	0.069	0.02	0.01	0.940	<0.01
Low frequency of fast-food consumption	4.04	0.008	0.03	0.08	0.776	<0.01
Low-fat food consumption	2.29	0.078	0.02	1.52	0.219	<0.01
Whole-grain consumption	2.09	0.102	0.02	1.8	0.180	<0.01
Plant-based protein consumption	0.66	0.576	0.01	2.71	0.100	0.01
Low frequency of leafy green vegetable intake	4.95	0.002	0.04	12.12	<0.001	0.03
Daily grain servings	1.75	0.156	0.01	18.18	<0.001	0.05
Daily vegetable servings	1.51	0.211	0.01	1.37	0.242	<0.01
Daily fruit servings	2.01	0.111	0.02	0.78	0.376	<0.01
Daily dairy servings	0.66	0.577	0.01	17.82	<0.001	0.04
Daily protein servings	0.18	0.91	<0.01	3.77	0.053	0.01
Daily sweets and dessert servings	4.13	0.007	0.03	5.56	0.019	0.01
Daily fat servings	1.13	0.336	0.01	0.41	0.521	<0.01
Frequency of salt addition or salty food consumption	4.85	0.003	0.04	2.29	0.131	0.01
Daily water intake (glasses)	1.48	0.219	0.01	2.54	0.112	0.01
Current weight relative to 10 years ago (lower weight)	0.33	0.804	<0.01	0.08	0.775	<0.01
Calcium supplementation (no use)	5.28	0.001	0.04	24.02	<0.001	0.06
Absence of dieting behavior	2.3	0.077	0.02	0.13	0.718	<0.01
Daily caffeine intake	2.29	0.078	0.02	12.43	<0.001	0.03
Alcohol consumption	1.59	0.19	0.01	0.5	0.479	<0.01
Smoking behavior	2.25	0.082	0.02	0.35	0.556	<0.01
BMI	0.698	0.553	0.01	5.059	0.025	0.01

## References

[1] Owen L., Corfe B. (2017). The Role of Diet and Nutrition on Mental Health and Wellbeing. Proc. Nutr. Soc..

[2] Suárez-López L.M., Bru-Luna L.M., Martí-Vilar M. (2023). Influence of Nutrition on Mental Health: Scoping Review. Healthcare.

[3] Bodnar L.M., Wisner K.L. (2005). Nutrition and Depression: Implications for Improving Mental Health among Childbearing-Aged Women. Biol. Psychiatry.

[4] Boyle N., Lawton C., Dye L. (2017). The Effects of Magnesium Supplementation on Subjective Anxiety and Stress—A Systematic Review. Nutrients.

[5] Parker G.B., Brotchie H., Graham R.K. (2017). Vitamin D and Depression. J. Affect. Disord..

[6] Andreo-Martínez P., García-Martínez N., Sánchez-Samper E.P. (2018). Gut Microbiota and Its Relationship with Mental Illness through the Microbiota–Gut–Brain Axis. J. Clin. Neurosci..

[7] Lange K.W., Lange K.M., Nakamura Y., Kanaya S. (2020). Is There a Role of Gut Microbiota in Mental Health?. J. Food Bioact..

[8] Tao H., Wang C.-R., Guo J.-C., Guo M. (2020). Research Progress on the Relationship between Intestinal Flora and Mental and Psychological Diseases. Adv. Microbiol..

[9] Araiza A.M., Lobel M. (2018). Stress and Eating: Definitions, Findings, Explanations, and Implications. Soc. Personal. Psychol. Compass.

[10] Hill D., Conner M., Clancy F., Moss R., Wilding S., Bristow M., O’Connor D.B. (2022). Stress and Eating Behaviours in Healthy Adults: A Systematic Review and Meta-Analysis. Health Psychol. Rev..

[11] Mikolajczyk R.T., El Ansari W., Maxwell A.E. (2009). Food Consumption Frequency and Perceived Stress and Depressive Symptoms among Students in Three European Countries. Nutr. J..

[12] Newman E., O’Connor D.B., Conner M. (2007). Daily Hassles and Eating Behaviour: The Role of Cortisol Reactivity Status. Psychoneuroendocrinology.

[13] O’Connor D.B., Jones F., Conner M., McMillan B., Ferguson E. (2008). Effects of Daily Hassles and Eating Style on Eating Behavior. Health Psychol..

[14] Roberts C.J., Campbell I.C., Troop N. (2014). Increases in Weight during Chronic Stress Are Partially Associated with a Switch in Food Choice towards Increased Carbohydrate and Saturated Fat Intake. Eur. Eat. Disord. Rev..

[15] Wallis D.J., Hetherington M.M. (2009). Emotions and Eating. Self-Reported and Experimentally Induced Changes in Food Intake under Stress. Appetite.

[16] Adam T.C., Epel E.S. (2007). Stress, Eating and the Reward System. Physiol. Behav..

[17] Blanchflower D.G., Oswald A.J., Stewart-Brown S. (2013). Is Psychological Well-Being Linked to the Consumption of Fruit and Vegetables?. Soc. Indic. Res..

[18] Conner T.S., Brookie K.L., Richardson A.C., Polak M.A. (2015). On Carrots and Curiosity: Eating Fruit and Vegetables Is Associated with Greater Flourishing in Daily Life. Br. J. Health Psychol..

[19] Grant N., Wardle J., Steptoe A. (2009). The Relationship between Life Satisfaction and Health Behavior: A Cross-Cultural Analysis of Young Adults. Int. J. Behav. Med..

[20] Rooney C., McKinley M.C., Woodside J.V. (2013). The Potential Role of Fruit and Vegetables in Aspects of Psychological Well-Being: A Review of the Literature and Future Directions. Proc. Nutr. Soc..

[21] De Vos J.A., Radstaak M., Bohlmeijer E.T., Westerhof G.J. (2018). Having an Eating Disorder and Still Being Able to Flourish? Examination of Pathological Symptoms and Well-Being as Two Continua of Mental Health in a Clinical Sample. Front. Psychol..

[22] Fowler K., Wareham-Fowler S., Keyes C.L.M. (2025). Flourishing despite Eating Disorder Risk: Exploring Undergraduate Student Academic, Social and Mental Health Outcomes via the Dual Continua Model. Psychol. Health Med..

[23] Gibson E.L. (2012). The Psychobiology of Comfort Eating: Implications for Neuropharmacological Interventions. Behav. Pharmacol..

[24] Greeno C.G., Wing R.R. (1994). Stress-Induced Eating. Psychol. Bull..

[25] Tomiyama A.J. (2019). Stress and Obesity. Annu. Rev. Psychol..

[26] Bandura A. (1997). Self-Efficacy: The Exercise of Control.

[27] Anderson E.S., Winett R.A., Wojcik J.R. (2000). Social-Cognitive Determinants of Nutrition Behavior among Supermarket Food Shoppers: A Structural Equation Analysis. Health Psychol..

[28] Anderson E.S., Winett R.A., Wojcik J.R., Williams D.M. (2010). Social Cognitive Mediators of Change in a Group Randomized Nutrition and Physical Activity Intervention: Social Support, Self-Efficacy, Outcome Expectations and Self-Regulation in the Guide-to-Health Trial. J. Health Psychol..

[29] Fredrickson B.L. (2004). The Broaden–and–Build Theory of Positive Emotions. Phil. Trans. R. Soc. Lond. B.

[30] Fritz M.M., Armenta C.N., Walsh L.C., Lyubomirsky S. (2019). Gratitude Facilitates Healthy Eating Behavior in Adolescents and Young Adults. J. Exp. Soc. Psychol..

[31] Worthington E.L., Scherer M. (2007). Forgiveness Is an Emotion-Focused Coping Strategy That Can Reduce Health Risks and Promote Health Resilience: Theory, Review, and Hypotheses. Psychol. Health.

[32] Skalski-Bednarz S.B., Toussaint L.L., Konaszewski K., Surzykiewicz J. (2024). Personality Traits as Predictors of Forgiveness and Gratitude/Awe: A Two-Wave Longitudinal Study. Curr. Psychol..

[33] Toussaint L.L., Seawell A.H., Elder K., Surzykiewicz J., Skalski-Bednarz S.B. (2026). Forgiveness and My Waistline: Emotional Eating Mediates the Relationship between Forgiveness and Adult Weight Gain. J. Public Health.

[34] Oman D., Oman D. (2018). Public Health Nutrition, Religion, and Spirituality. Why Religion and Spirituality Matter for Public Health.

[35] Park C.L. (2007). Religiousness/Spirituality and Health: A Meaning Systems Perspective. J. Behav. Med..

[36] VanderWeele T.J. (2017). Religious Communities and Human Flourishing. Curr. Dir. Psychol. Sci..

[37] Falb M.D., Pargament K.I., Teramoto Pedrotti J., Edwards L.M. (2014). Religion, Spirituality, and Positive Psychology: Strengthening Well-Being. Perspectives on the Intersection of Multiculturalism and Positive Psychology.

[38] Tan M.-M., Chan C.K.Y., Reidpath D.D. (2013). Religiosity and Spirituality and the Intake of Fruit, Vegetable, and Fat: A Systematic Review. Evid.-Based Complement. Altern. Med..

[39] Salim I., Al Suwaidi J., Ghadban W., Alkilani H., Salam A.M. (2013). Impact of Religious Ramadan Fasting on Cardiovascular Disease: A Systematic Review of the Literature. Curr. Med. Res. Opin..

[40] Chouraqui J.-P., Turck D., Briend A., Darmaun D., Bocquet A., Feillet F., Frelut M.-L., Girardet J.-P., Guimber D., Hankard R. (2021). Religious Dietary Rules and Their Potential Nutritional and Health Consequences. Int. J. Epidemiol..

[41] Akrawi D., Bartrop R., Potter U., Touyz S. (2015). Religiosity, Spirituality in Relation to Disordered Eating and Body Image Concerns: A Systematic Review. J. Eat. Disord..

[42] Richards P.S., Weinberger-Litman S.L., Susov S., Berrett M.E., Pargament K.I., Mahoney A., Shafranske E.P. (2013). Religiousness and Spirituality in the Etiology and Treatment of Eating Disorders. APA Handbook of Psychology, Religion, and Spirituality: An Applied Psychology of Religion and Spirituality.

[43] Sandage S.J., Shults F.L. (2007). Relational Spirituality and Transformation: A Relational Integration Model. J. Psychol. Christ..

[44] Tomlinson J., Glenn E.S., Paine D.R., Sandage S.J. (2016). What Is the “Relational” in Relational Spirituality? A Review of Definitions and Research Directions. J. Spiritual. Ment. Health.

[45] Cohen S., Kamarck T., Mermelstein R. (1983). A Global Measure of Perceived Stress. J. Health Soc. Behav..

[46] Radloff L.S. (1977). The CES-D Scale. Appl. Psychol. Meas..

[47] Chesney M.A., Neilands T.B., Chambers D.B., Taylor J.M., Folkman S. (2006). A Validity and Reliability Study of the Coping Self-efficacy Scale. Br. J. Health Psychol..

[48] Underwood L.G., Teresi J.A. (2002). The Daily Spiritual Experience Scale: Development, Theoretical Description, Reliability, Exploratory Factor Analysis, and Preliminary Construct Validity Using Health-Related Data. Ann. Behav. Med..

[49] Fetzer Institute (1999). Multidimensional Measurement of Religiousness/Spirituality for Use in Health Research.

[50] McCullough M.E., Emmons R.A., Tsang J.-A. (2002). The Grateful Disposition: A Conceptual and Empirical Topography. J. Personal. Soc. Psychol..

[51] Brown R.P. (2003). Measuring Individual Differences in the Tendency to Forgive: Construct Validity and Links with Depression. Pers. Soc. Psychol. Bull..

[52] Parklawn Federal Occupational Health Unit (2000). Personal Wellness Profile.

[53] Marsh H.W., Lüdtke O., Trautwein U., Morin A.J.S. (2009). Classical Latent Profile Analysis of Academic Self-Concept Dimensions: Synergy of Person- and Variable-Centered Approaches to Theoretical Models of Self-Concept. Struct. Equ. Model. Multidiscip. J..

[54] Lu B., Ma L., Xia F., Zhang L., Liu Y., Yu X., Sun R., Luo Y. (2026). Flourishing and Its Influencing Factor in Inflammatory Bowel Disease Patients: A Latent Profile Analysis. Front. Psychiatry.

[55] Davis E.B., Chen Z.J., Cowden R.G., VanderWeele T.J., Oberg S., Bivins G., Newcity C., Song E., Koenig H.G. (2026). An Overview of Systematic Reviews and Meta-Analyses on the Association between Religion/Spirituality and Health/Well-Being. Handbook of Spirituality, Health, and Well-Being.

[56] Masters R.A., Berkeley C. (2010). Spiritual Bypassing: When Spirituality Disconnects Us from What Really Matters.

[57] Tepper L., Rogers S.A., Coleman E.M., Malony H.N. (2001). The Prevalence of Religious Coping among Persons with Persistent Mental Illness. Psychiatr. Serv..

[58] Sandage S.J., Williamson I. (2010). Relational Spirituality and Dispositional Forgiveness: A Structural Equations Model. J. Psychol. Theol..

[59] Keyes C.L.M. (2005). Mental Illness and/or Mental Health? Investigating Axioms of the Complete State Model of Health. J. Consult. Clin. Psychol..

[60] Skalski-Bednarz S.B., Hillert A., Surzykiewicz J., Riedl E., Harder J.-P., Hillert S.M., Adamczyk M., Uram P., Konaszewski K., Rydygel M. (2024). Longitudinal Impact of Disordered Eating Attitudes on Depression, Anxiety, and Somatization in Young Women with Anorexia and Bulimia. J. Clin. Med..

[61] Afshin A., Sur P.J., Fay K.A., Cornaby L., Ferrara G., Salama J.S., Mullany E.C., Abate K.H., Abbafati C., Abebe Z. (2019). Health Effects of Dietary Risks in 195 Countries, 1990–2017: A Systematic Analysis for the Global Burden of Disease Study 2017. Lancet.

[62] World Health Organization Healthy Diet. https://www.who.int/news-room/fact-sheets/detail/healthy-diet.

[63] Shallu P.B. (2021). Efficacy of Gratitude and Forgiveness as Positive Psychological Intervention in Reducing Depression. IAHRW Int. J. Soc. Sci..

[64] Vos J., Vitali D. (2018). The Effects of Psychological Meaning-Centered Therapies on Quality of Life and Psychological Stress: A Metaanalysis. Palliat. Support. Care.

[65] Dean M., Raats M.M., Grunert K.G., Lumbers M., Team T.F. (2009). in L.L. Factors Influencing Eating a Varied Diet in Old Age. Public. Health Nutr..

[66] Jovičić A.Đ. (2015). Healthy Eating Habits among the Population of Serbia: Gender and Age Differences. J. Health Popul. Nutr..

[67] Folch C. (2010). Stimulating Consumption: Yerba Mate Myths, Markets, and Meanings from Conquest to Present. Comp. Stud. Soc. Hist..

[68] Hladun O., Papaseit E., Martín S., Barriocanal A.M., Poyatos L., Farré M., Pérez-Mañá C. (2021). Interaction of Energy Drinks with Prescription Medication and Drugs of Abuse. Pharmaceutics.

[69] Buecker S., Luhmann M., Haehner P., Bühler J.L., Dapp L.C., Luciano E.C., Orth U. (2023). The Development of Subjective Well-Being across the Life Span: A Meta-Analytic Review of Longitudinal Studies. Psychol. Bull..

[70] Smith J., Borchelt M., Maier H., Jopp D. (2002). Health and Well–Being in the Young Old and Oldest Old. J. Soc. Issues.

[71] Worthington E.L. (2024). Forgiveness Psychoeducation with Emerging Adults: Forgiveness and Community Campaigns for Forgiveness. Educ. Sci..

[72] Pew Research Center (2024). Religion’s Role in Public Life. https://www.pewresearch.org/religion/2024/03/15/religions-role-in-public-life/.

